# Association between absolute lymphocyte count and outcomes of radiotherapy combined with immunotherapy in elderly patients with inoperable esophageal squamous cell carcinoma

**DOI:** 10.3389/fonc.2026.1953153

**Published:** 2026-10-23

**Authors:** Youqin Jiang, Weiwei Chen, Daguang Ni, Xu Liang

**Affiliations:** 1 Department of Radiotherapy, Yancheng Third People’s Hospital, The affiliated hospital of Jiangsu Medical College, Yancheng, China; 2 Department of Radiotherapy, The Sixth Affiliated Hospital of Nantong University, Yancheng Third People’s Hospital, Yancheng, China

**Keywords:** absolute lymphocyte count, aged, esophageal squamous cell carcinoma, immunotherapy, prognosis, radiotherapy

## Abstract

**Objective:**

To determine whether pretreatment absolute lymphocyte count (ALC) is related to clinical outcomes among elderly patients with inoperable ESCC receiving definitive radiotherapy combined with immunotherapy.

**Methods:**

Elderly patients (≥ 60 years) with incurable ESCC who underwent programmed death-1 (PD-1) inhibitor therapy and definitive radiotherapy were retrospectively enrolled. ROC analysis identified the most discriminative ALC cutoff. Short-term efficacy was compared between groups, and Cox regression assessed factors related to overall survival (OS) and progression-free survival (PFS).

**Results:**

Data from 102 elderly patients were evaluated. The objective response rate (ORR) was 70.59%; median PFS and OS were 21 and 28 months, respectively. ROC analysis identified 1.24 × 10^9^/L as the cutoff value for ALC. The response rate was markedly higher among patients with high ALC than among those with low ALC (86.0% vs. 51.1%, *P* < 0.001). ALC was identified as an independent factor associated with short-term treatment response by multivariate logistic regression (OR = 4.716, 95% CI: 1.167–19.062, *P* = 0.030). Longer median OS and PFS were observed in high-ALC patients. In the multivariate Cox analysis, higher ALC remained independently related to improved PFS (HR = 0.450, 95% CI 0.213–0.951, P = 0.036) and improved OS (HR = 0.425, 95% CI 0.184–0.982, *P* = 0.045).

**Conclusion:**

Baseline ALC has the potential to be used to guide patient selection and individualized treatment planning, because this readily available biomarker was independently associated with treatment response and survival.

## Introduction

Esophageal cancer continues to impose a substantial global health burden because of its high incidence and mortality. Worldwide, more than 600,000 new cases are diagnosed and over 540,000 deaths occur each year, with a disproportionately high burden in East Asia, according to GLOBOCAN 2020. In China, esophageal squamous cell carcinoma (ESCC) constitutes more than 90% of esophageal cancer cases, which presents a significant public health concern (1, 2). For many patients who cannot undergo surgery, non-surgical treatment represents the main therapeutic approach. Although definitive chemoradiotherapy (dCRT) has long been considered as a standard treatment for locally advanced inoperable ESCC, results remain unsatisfactory. In the RTOG 85–01 trial, the 2- and 5-year overall survival rates were only 36% and 26%, respectively (3). Furthermore, RTOG 94–05 demonstrated that increasing the radiation dose did not improve survival (4). Treatment is particularly challenging in elderly patients, who often have comorbidities, poor nutritional status, and limited physiological reserve (5). These factors frequently preclude surgery or intensive chemotherapy. Although radiotherapy is generally better tolerated, long-term outcomes in this population remain unsatisfactory, indicating a need for more effective treatment approaches.

The treatment of ESCC has changed considerably with the introduction of immune checkpoint inhibitors (ICIs). Immunotherapy-based combinations have become an important standard treatment option for advanced ESCC since Phase III trials like CheckMate 648 and ESCORT-1st demonstrated that immunotherapy plus chemotherapy dramatically increased survival when compared to traditional regimens (6, 7). Definitive radiotherapy remains a key treatment option for patients with inoperable ESCC. Mounting evidence suggests that radiation therapy may work in concert with ICIs by enhancing T-cell infiltration, increasing PD-L1 expression, and promoting antigen presentation (8). Several studies have reported that radiotherapy combined with ICIs is an effective and tolerable strategy for advanced ESCC (9, 10). These findings suggest that radiotherapy-based immunotherapy may offer potential clinical benefit to elderly patients who are unsuitable for surgery, although better methods are still needed to identify patients who may benefit most.

Prognosis, treatment response, and tumor progression are all strongly correlated with chronic inflammation, which is acknowledged as a characteristic of cancer. Inflammation-related markers obtained from routine blood tests, such as the neutrophil-to-lymphocyte ratio (NLR), platelet-to-lymphocyte ratio (PLR), and lymphocyte-to-monocyte ratio (LMR), have been linked to clinical outcomes in cancers, including ESCC (11, 12). Absolute lymphocyte count (ALC), a readily available indicator of host immune status, may be linked to treatment outcomes, according to emerging data. A previous study found that pretreatment ALC was independently associated with pathological response and progression-free survival (PFS) among patients with ESCC receiving neoadjuvant chemoradiotherapy and immunotherapy (13). Similar associations between ALC or related inflammatory markers and clinical outcomes have also been observed in other tumor types treated with immunotherapy (14, 15). However, its prognostic value in elderly patients undergoing definitive radiotherapy combined with immunotherapy for ESCC remains unclear.

We retrospectively examined elderly patients with inoperable ESCC who received radiotherapy plus immunotherapy to explore factors related to treatment response and prognosis. Pretreatment ALC received special attention because of its accessibility and ability to represent systemic immune status. The results may provide further insight into host immune features in this context and offer initial evidence in favor of more customized treatment approaches for older ESCC patients.

## Materials and methods

### Study population

Consecutive elderly patients with inoperable ESCC treated at Yancheng Third People’s Hospital from June 2020 to June 2024 were included in this retrospective analysis. All patients received radiotherapy and immunotherapy. Eligibility criteria included an age of at least 60 years, a performance status (PS) score of 0 or 1, and histologically verified ESCC. Inoperability was determined based on local invasion, distant metastasis, or involvement of critical structures on imaging, or patients who met surgical indications but refused surgery for personal reasons (16). The patients were also required to have no prior history of antitumor therapy. Patients with previous malignancies, severe liver or kidney dysfunction, contraindications to radiotherapy or immunotherapy, or who had received hematopoietic agents before treatment were excluded.

Ethical approval was obtained from the Ethics Committee of Yancheng Third People’s Hospital (approval No. Lun-Shen-2025-96), and the study was conducted in compliance with the Declaration of Helsinki. Informed consent was waived due to the retrospective design of the study.

### Treatment

Tumor stage was determined using the 8th edition of the American Joint Committee on Cancer (AJCC) TNM classification (17). Radiotherapy was given at a total dose of 50.4–61.2 Gy in 1.8–2.0 Gy fractions once daily, five days per week, using intensity-modulated radiation therapy (IMRT) with 6 MV photon beams. The dose limits for normal tissues were as follows: lung V20 was limited to ≤ 30%, mean lung dose ≤15 Gy, heart V40 to ≤ 30% and V30 to ≤ 40%, and spinal cord Dmax ≤ 45 Gy. Camrelizumab immunotherapy (200 mg every three weeks) was administered concurrently with or sequentially after radiotherapy. Chemotherapy was prescribed based on patients’ conditions and tolerance and included either no chemotherapy, oral S-1 concurrently, or intravenous paclitaxel plus cisplatin (TP) regimen concurrently with radiotherapy.

### Data collection

Baseline demographic and clinical information was extracted from medical records, including sex and age, PS score, BMI, serum albumin level, history of smoking and alcohol consumption, tumor site, lesion length, clinical stage, chemotherapy received, immunotherapy timing, and number of immunotherapy cycles. Complete blood count (CBC) within a week prior to treatment, including ANC, ALC, AMC, and PLT, was recorded. The NLR was defined as ANC/ALC, the MLR as AMC/ALC, and the PLR as PLT/ALC (18). Imaging and endoscopic evaluation included chest CT, esophagography, and upper gastrointestinal endoscopy performed within 1 month before treatment.

### Outcome assessment

During the first two years after radiotherapy, follow-up was scheduled at three-month intervals; from years three to five, patients were monitored every six months. Vital signs, physical examination, upper gastrointestinal endoscopy, esophagography, chest CT, and laboratory testing were among the follow-up evaluations. Follow-up ended on June 30, 2025. The short-term therapeutic response was assessed three months after therapy according to the Response Evaluation Criteria in Solid Tumors (RECIST) version 1.1 (19). Treatment efficacy was evaluated as complete response (CR), partial response (PR), stable disease (SD), or progressive disease (PD). Treatment response was defined as CR or PR, whereas SD or PD indicated non-response. The primary study outcomes were defined as follows: ORR was defined as the percentage of patients who achieved CR or PR. PFS was calculated from the start of treatment to confirmed disease progression or death from any cause. OS was calculated from the start of treatment to death from any cause.

### Statistical analysis

Descriptive statistics were applied to summarize baseline clinical characteristics. Continuous variables were presented as mean ± standard deviation (SD) and compared using the independent-samples *t* test, while categorical variables were presented as number and percentage and compared using the χ² test or Fisher’s exact test when appropriate. ROC curve analysis was conducted to identify the optimal ALC cutoff for distinguishing responders from non-responders, with the Youden index used as the selection criterion.

Univariate logistic regression was conducted first to assess variables related to short-term treatment response, and variables with *P* < 0.05 were subsequently included in the multivariate model. Survival outcomes were analyzed by the Kaplan-Meier method, and group differences were evaluated using the log-rank test. The reverse Kaplan-Meier method provided the estimate of median follow-up duration. Univariate Cox proportional hazards models were constructed to examine potential prognostic factors for PFS and OS. Multivariate Cox regression was then used for variables that reached statistical significance. ALC was analyzed continuously in both logistic and Cox regression models. Bootstrap analysis with 1,000 resamples was used to assess the stability of the ROC and multivariate logistic regression results. Exploratory subgroup and interaction analyses were performed according to chemotherapy regimen and immunotherapy timing. SPSS version 27.0 (IBM Corp., Armonk, NY, USA) and R version 4.4.0 (R Foundation for Statistical Computing, Vienna, Austria) were used for statistical analyses, whereas GraphPad Prism version 10.0 (GraphPad Software, San Diego, CA, USA) was used to generate ROC and survival curves. A two-sided *P* value <0.05 indicated statistical significance.

## Results

### Baseline characteristics

Overall, 102 patients were enrolled in this study, with a mean patient age of 75.1 years (60–88); 63.7% were male. Smoking and alcohol use were documented in 55.9% and 33.3%, respectively. PS scores were 0 in 56.9% of patients and 1 in 43.1%. Disease stages I–II and III–IV accounted for 39.2% and 60.8% of patients, respectively. 27.5% of tumors were found in the cervical or upper thoracic esophagus, while 72.5% were found in the middle/lower thoracic region. Mean serum albumin was 40.84 g/L, mean lesion length was 5.17 cm, and the mean BMI was 22.36. 23.5% of patients received no chemotherapy, 31.4% received S-1, and 45.1% received TP. The median number of immunotherapy cycles was 7 (1–29), with 19.6% receiving sequential and 80.4% receiving concurrent immunotherapy.

### Determination of the ALC cutoff value and comparison of clinical characteristics between groups

ROC analysis using the Youden index identified 1.24 × 10^9^/L as the optimal ALC cutoff for distinguishing responders from non-responders among elderly patients receiving radiotherapy and immunotherapy for inoperable ESCC. The AUC reached 0.708 (*P* = 0.001; 95% CI: 0.595–0.821); sensitivity and specificity were 68.06% and 73.33% (**Figure 1**). To assess the stability of the ROC results and the identified cutoff, bootstrap resampling was performed. The bootstrap analysis showed a median AUC of 0.709 (95% interval: 0.589–0.818) and a median optimal ALC cutoff of 1.185 × 10^9^/L (95% interval: 0.900–1.400 × 10^9^/L). The bootstrap-derived cutoff was close to the original estimate, and the latter fell within the bootstrap interval. Patients were classified into a high-ALC group (>1.24 × 10^9^/L) and a low-ALC group (≤1.24 × 10^9^/L) based on the original ROC-derived cutoff. A significantly higher ORR was observed in the high-ALC group than in the low-ALC group (86.0% vs. 51.1%, *P* < 0.001). Statistical comparisons of sex, age, smoking or alcohol history, performance status score, BMI, serum albumin, tumor location or length, disease stage, receipt of chemotherapy, immunotherapy timing, and number of immunotherapy cycles did not reach significance (**Table 1**).

**Figure 1:**
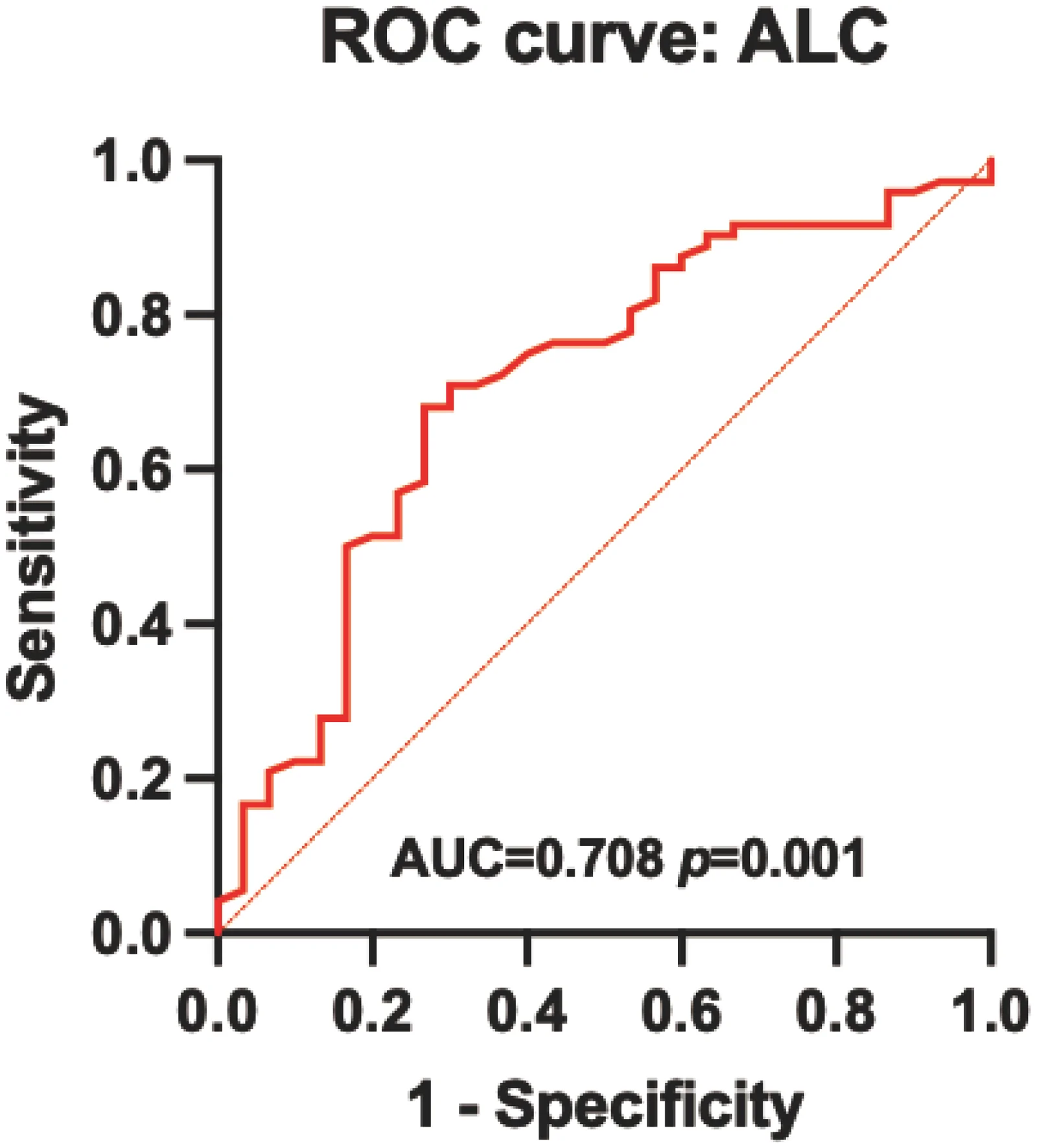
ROC curve of ALC for short-term treatment response in elderly inoperable ESCC.

**Table 1:** Baseline clinical characteristics of patients with high vs. low ALC.

General information	Low ALC group (≤1.24 × 10^9^/L)	High ALC group (>1.24 × 10^9^/L)	*P*
Age (years)	74.76 ± 5.89	74.89 ± 6.81	0.914
Gender			
Female	16 (35.6%)	21 (36.8%)	0.893
Male	29 (64.4%)	36 (63.2%)	
Smoking history			
No	17 (37.8%)	28 (49.1%)	0.252
Yes	28 (62.2%)	29 (50.9%)	
Drinking history			
No	30 (66.7%)	38 (66.7%)	1.000
Yes	15 (33.3%)	19 (33.3%)	
BMI (kg/m^2^)	22.55 ± 3.95	22.21 ± 3.57	0.656
Serum Albumin (g/L)	40.47 ± 4.13	41.13 ± 3.35	0.372
PS score			
0	25 (55.6%)	33 (57.9%)	0.813
1	20 (44.4%)	24 (42.1%)	
Tumor location			
Cervical + Upper	13 (28.9%)	15 (26.3%)	0.772
Middle + Lower	32 (71.1%)	42 (73.7%)	
Tumor Length (cm)	5.12 ± 2.34	5.31 ± 2.08	0.674
Stage			
I + II	16 (35.6%)	24 (42.1%)	0.501
III + IV	29 (64.4%)	33 (57.9%)	
Chemotherapy			
No	15 (33.3%)	9 (15.8%)	0.111
S-1	13 (28.9%)	19 (33.3%)	
TP	17 (37.8%)	29 (50.9%)	
Immunotherapy			
Sequential	9 (20%)	11 (19.3%)	0.929
Concurrent	36 (80%)	46 (80.7%)	
ICI cycle			
≤4	19 (42.2%)	27 (47.4%)	0.604
>4	26 (57.8%)	30 (52.6%)	
Response			
CR + PR	23 (51.1%)	49 (86%)	**<0.001**
SD + PD	22 (48.9%)	8 (14%)	

ALC, absolute lymphocyte count, and the cutoff value was 1.24 × 10^9^/L. PS, performance status; S-1, tegafur–gimeracil–oteracil; BMI, body mass index; TP, paclitaxel plus cisplatin; ICIs, immune checkpoint inhibitors; PR, partial response; CR, complete response; SD, stable disease; PD, progressive disease. No adjustment was made for multiple comparisons.

P-values < 0.05 are shown in bold.

### Factors related to short-term treatment response in elderly patients with inoperable ESCC

An overall ORR of 70.59% was obtained from the 102 patients, of whom 72 showed complete or partial response and formed the response group, while 30 showed stable or progressive disease and formed the non-response group. In univariate analysis, serum albumin level (*P* = 0.025), tumor stage (*P* = 0.038), ALC (*P* = 0.002), and PLR (*P* = 0.022) were related to short-term treatment response. Age, sex, smoking status, alcohol consumption, PS score, BMI, tumor site, lesion length, chemotherapy received, immunotherapy timing, NLR, and MLR showed no significant differences between the response and non-response groups (**Table 2**). In the multivariate model, ALC (OR = 4.716, 95% CI: 1.167–19.062, *P* = 0.030) and tumor stage (OR = 0.337, 95% CI: 0.120–0.951, *P* = 0.040) retained independent associations with short-term treatment response in this patient population (**Table 3**). These associations were further examined using bootstrap analysis. Both ALC (*P* = 0.033, 95% CI for B: 0.129–3.638) and tumor stage (*P* = 0.035, 95% CI for B: −2.446 to −0.095) remained significantly associated with treatment response.

**Table 2:** Clinical characteristics by treatment response.

General information	Response (PR + CR) n=72	No response (SD + PD) n=30
Age (years)	74.46 ± 6.29	75.73 ± 6.64
Gender		
Female	28 (38.9%)	9 (30.0%)
Male	44 (61.1%)	21 (70.0%)
Smoking history		
No	33 (45.8%)	12 (40.0%)
Yes	39 (54.2%)	18 (60.0%)
Drinking history		
No	51 (70.8%)	17 (56.7%)
Yes	21 (29.2%)	13 (43.3%)
PS score		
0	43 (59.7%)	15 (50.0%)
1	29 (40.3%)	15 (50.0%)
BMI (kg/m^2^)	22.63 ± 4.03	21.71 ± 2.84
Serum Albumin (g/L)	41.39 ± 3.56	39.52 ± 3.77
Tumor location		
Cervical + Upper	23 (31.9%)	5 (16.7%)
Middle + Lower	49 (68.1%)	25 (83.3%)
Tumor Length (cm)	5.19 ± 2.09	5.32 ± 2.45
Stage		
I + II	33 (45.8%)	7 (23.3%)
III + IV	39 (54.2%)	23 (76.7%)
Chemotherapy		
No	14 (19.5%)	10 (33.3%)
S-1	23 (31.9%)	9 (30.0%)
TP	35 (48.6%)	11 (36.7%)
Immunotherapy		
Sequential	12 (16.7%)	8 (26.7%)
Concurrent	60 (83.3%)	22 (73.3%)
ALC (× 10^9^/L)	1.41 ± 0.43	1.10 ± 0.42
NLR	3.14 ± 1.81	3.89 ± 1.85
MLR	0.57 ± 0.26	0.67 ± 0.48
PLR	139.13 ± 72.80	194.06 ± 137.17

Abbreviations are defined in **Table 1**. NLR, neutrophil-to-lymphocyte ratio; MLR, monocyte-to-lymphocyte ratio; PLR, platelet-to-lymphocyte ratio.

**Table 3:** Univariate and multivariate logistic regression of factors associated with short-term treatment response in elderly patients with inoperable ESCC.

General information	Univariate analysis		Multivariate analysis	
	OR, 95% CI	*P*	OR, 95% CI	*P*
Age (years)	0.968 (0.904–1.037)	0.358		
Gender				
Female	1			
Male	0.673 (0.270–1.679)	0.396		
Smoking history				
No	1			
Yes	0.788 (0.332–1.871)	0.589		
Drinking history				
No	1			
Yes	0.538 (0.223–1.302)	0.169		
PS score				
0	1			
1	0.674 (0.286–1.589)	0.368		
BMI (kg/m^2^)	1.075 (0.949–1.216)	0.256		
Serum Albumin (g/L)	1.152 (1.018–1.303)	**0.025**	1.111 (0.968–1.275)	0.133
Tumor location				
Cervical + Upper	1			
Middle + Lower	0.426 (0.145–1.255)	0.122		
Tumor Length (cm)	0.973 (0.801–1.182)	0.785		
Stage				
I + II	1		1	
III + IV	0.360 (0.137–0.944)	**0.038**	0.337 (0.120–0.951)	**0.040**
Chemotherapy				
No	1			
S-1	1.825 (0.596–5.590)	0.292		
TP	2.273 (0.790–6.541)	0.128		
Immunotherapy				
Sequential	1			
Concurrent	1.818 (0.656–5.039)	0.250		
ALC (× 10^9^/L)	5.815 (1.870–18.079)	**0.002**	4.716 (1.167–19.062)	**0.030**
NLR	0.809 (0.644–1.016)	0.068		
MLR	0.430 (0.126–1.461)	0.176		
PLR	0.995 (0.990–0.999)	**0.022**	0.999 (0.993–1.005)	0.795

Abbreviations are defined in Tables 1 and 2. OR, odds ratio; CI, confidence interval. Treatment response was coded as 1 and non-response as 0. P-values < 0.05 are shown in bold.

### Prognostic significance of ALC for PFS and OS in elderly patients with inoperable ESCC

A total of 67 PFS events and 55 deaths were recorded. Follow-up duration had a median of 33 months (95% CI: 28.2–37.8 months). For PFS, univariate Cox analysis identified disease stage (HR = 2.347, 95% CI: 1.379–3.994, *P* = 0.002), ALC (HR = 0.414, 95% CI: 0.225–0.764, *P* = 0.005), and PLR (HR = 1.003, 95% CI: 1.000–1.005, *P* = 0.031) as significant prognostic factors. Following multivariate adjustment, stage III–IV (HR = 2.457, 95% CI: 1.437–4.200, *P* = 0.001), and ALC (HR = 0.450, 95% CI: 0.213–0.951, *P* = 0.036) remained independently related to PFS (**Table 4**). For OS (**Table 5**), univariate Cox analysis showed that disease stage (HR = 2.317, 95% CI: 1.285–4.175, *P* = 0.005), ALC (HR = 0.359, 95% CI: 0.178–0.724, *P* = 0.004), and PLR (HR = 1.004, 95% CI: 1.001–1.006, *P* = 0.005) were related to OS. After multivariate adjustment, stage III–IV (HR = 2.592, 95% CI: 1.427–4.708, *P* = 0.002), and ALC (HR = 0.425, 95% CI: 0.184–0.982, *P* = 0.045) remained statistically significant.

**Table 4:** Univariate and multivariate Cox regression for PFS.

General information	PFS			
	Univariate analysis		Multivariate analysis	
	HR, 95% CI	*P*	HR, 95% CI	*P*
Age (years)				
60–69	1			
70–79	1.434 (0.755–2.722)	0.271		
≥80	1.427 (0.685–2.973)	0.342		
Gender				
Female	1			
Male	1.019 (0.615–1.689)	0.942		
Smoking history				
No	1			
Yes	1.338 (0.815–2.197)	0.250		
Drinking history				
No	1			
Yes	1.272 (0.773–2.092)	0.334		
PS score				
0	1			
1	1.095 (0.676–1.774)	0.712		
BMI (kg/m^2^)	0.954 (0.889–1.024)	0.190		
Serum Albumin (g/L)	0.982 (0.920–1.048)	0.580		
Tumor location				
Cervical + Upper	1			
Middle + Lower	1.470 (0.827–2.612)	0.189		
Tumor Length (cm)	1.058 (0.947–1.182)	0.320		
Stage				
I + II	1		1	
III + IV	2.347 (1.379–3.994)	**0.002**	2.457 (1.437–4.200)	**0.001**
Chemotherapy				
No	1			
S-1	0.773 (0.404–1.479)	0.437		
TP	0.659 (0.357–1.219)	0.184		
Immunotherapy				
Sequential	1			
Concurrent	0.834 (0.475–1.463)	0.527		
ALC (× 10^9^/L)	0.414 (0.225–0.764)	**0.005**	0.450 (0.213–0.951)	**0.036**
NLR	1.108 (0.985–1.245)	0.088		
MLR	2.034 (0.965–4.287)	0.062		
PLR	1.003 (1.000–1.005)	**0.031**	1.001 (0.998–1.004)	0.557

Abbreviations are defined in **Tables 1** and **2**. HR, hazard ratio; CI, confidence interval.

P-values < 0.05 are shown in bold.

**Table 5:** Univariate and multivariate Cox regression for OS.

General information	OS			
	Univariate analysis		Multivariate analysis	
	HR, 95% CI	*P*	HR, 95% CI	*P*
Age (years)				
60–69	1			
70–79	1.476 (0.715–3.048)	0.293		
≥80	1.577 (0.684–3.637)	0.285		
Gender				
Female	1			
Male	1.043 (0.598–1.822)	0.881		
Smoking history				
No	1			
Yes	1.069 (0.622–1.838)	0.809		
Drinking history				
No	1			
Yes	1.118 (0.640–1.953)	0.695		
PS score				
0	1			
1	1.112 (0.652–1.896)	0.696		
BMI (kg/m^2^)	0.932 (0.858–1.012)	0.094		
Serum Albumin (g/L)	0.964 (0.894–1.038)	0.332		
Tumor location				
Cervical + Upper	1			
Middle + Lower	1.610 (0.830–3.125)	0.159		
Tumor Length (cm)	1.045 (0.924–1.182)	0.483		
Stage				
I + II	1		1	
III + IV	2.317 (1.285–4.175)	**0.005**	2.592 (1.427–4.708)	**0.002**
Chemotherapy				
No	1			
S-1	0.661 (0.328–1.329)	0.245		
TP	0.511 (0.259–1.011)	0.054		
Immunotherapy				
Sequential	1			
Concurrent	0.974 (0.510–1.860)	0.937		
ALC (× 10^9^/L)	0.359 (0.178–0.724)	**0.004**	0.425 (0.184–0.982)	**0.045**
NLR	1.122 (0.986–1.277)	0.081		
MLR	1.613 (0.725–3.591)	0.241		
PLR	1.004 (1.001–1.006)	**0.005**	1.002 (0.999–1.005)	0.213

Abbreviations are defined in **Tables 1**, **2**, and **4**.

P-values < 0.05 are shown in bold.

Further exploratory subgroup analyses showed similar directions of association across chemotherapy regimens and immunotherapy timing. No significant interaction was found between ALC and chemotherapy regimen for either PFS or OS. The interaction between ALC and immunotherapy timing was also not significant for PFS. For OS, the interaction was not significant before adjustment (*P* for interaction = 0.125) but became significant after adjustment (adjusted *P* for interaction = 0.031) (**Supplementary Table S1**).

The median OS for the entire cohort was 28 months, whereas the median PFS was 21 months. Specifically, patients with ALC ≤ 1.24 × 10^9^/L had a median PFS of 12 months, compared with 27 months in those with ALC >1.24 × 10^9^/L (**Figure 2A**), and Kaplan-Meier analysis showed a significant between-group difference (*P* = 0.001; HR = 2.078, 95% CI: 1.250–3.457). Additionally, the median OS of the low-ALC group was 24 months, while that of the high-ALC group was 36 months (**Figure 2B**), with the survival curves differing significantly between the two groups (*P* = 0.005; HR = 2.040, 95% CI: 1.166–3.568).

**Figure 2:**
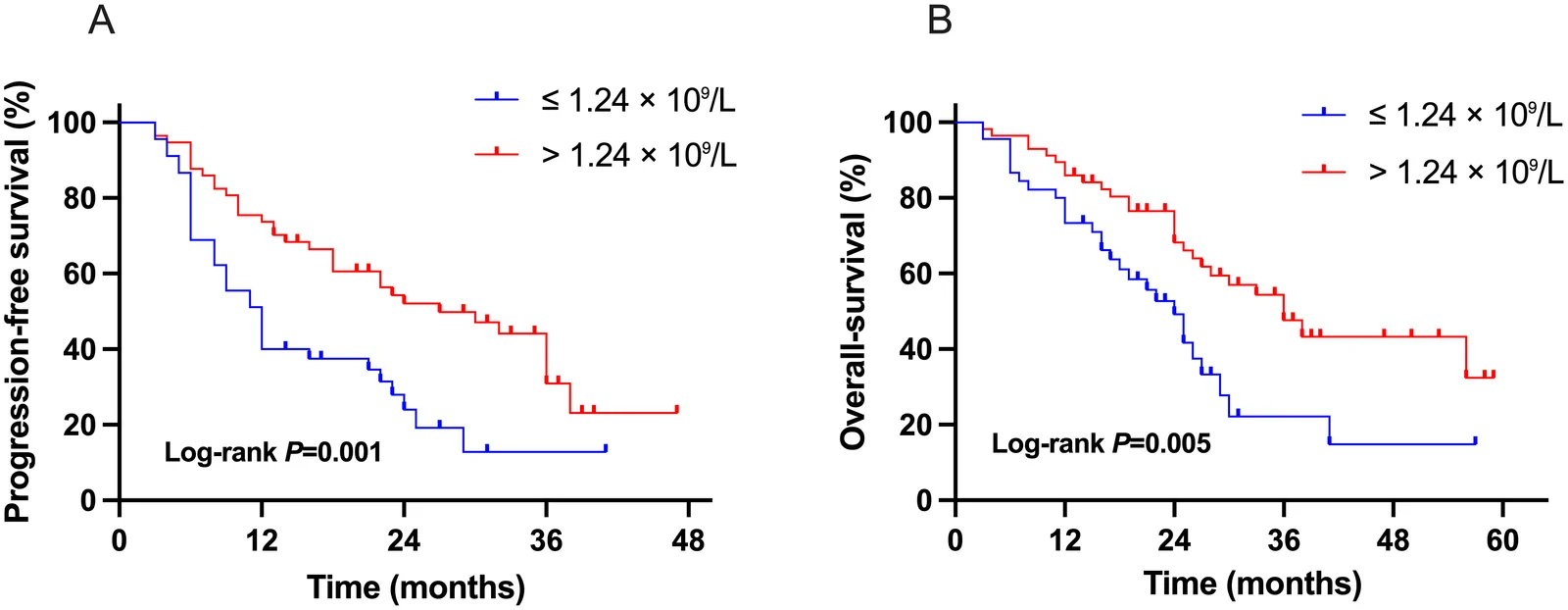
PFS **(A)** and OS **(B)** Curves by High vs. Low ALC in Elderly Inoperable ESCC Patients Treated with Radiotherapy Plus Immunotherapy.

## Discussion

This study explored the efficacy of definitive radiotherapy plus immunotherapy among elderly patients with inoperable ESCC and examined the prognostic value of baseline inflammatory markers. The combined treatment achieved an ORR of 70.59%, with PFS reaching a median of 21 months and OS reaching a median of 28 months. Among the evaluated inflammatory markers, baseline ALC was independently associated with short-term treatment response, PFS, and OS. Patients with higher baseline ALC showed better clinical outcomes.

Although elderly patients typically have reduced physiological reserve and multiple comorbidities, outcomes in this cohort were encouraging. The observed median OS of 28 months and ORR of 70.59% were higher than those reported in historical studies of elderly patients treated with definitive chemoradiotherapy alone, in whom median OS is generally reported at approximately 13–14 months (20). These findings are consistent with emerging evidence suggesting potential benefit from combining radiotherapy with immunotherapy, including a multicenter real-world study reporting a median PFS of 22.4 months in inoperable ESCC (21). Importantly, nearly half of ESCC patients are 70 years or older, yet elderly individuals often receive suboptimal regimens due to fears of toxicity or inferior immune responsiveness (22). Our results suggest that, in appropriately selected patients, elderly individuals may still achieve favorable outcomes with combined treatment approaches. This may reflect the importance of biological rather than chronological age in treatment decision-making, although this interpretation should be made cautiously. Radiotherapy may also contribute to these favorable outcomes through its effects on the immune system. It may promote tumor antigen release while also altering the tumor microenvironment, thereby potentially improving the response to immunotherapy (8).

Our findings further indicate that ALC is strongly associated with both treatment response and survival, with a more consistent association compared with other systemic inflammatory indices. ALC likely reflects baseline immune competence, which is essential for effective immunotherapy. Lymphocytes, particularly CD8^+^ T cells, are important for cancer cell killing and immune memory formation. When lymphocyte levels are reduced, the effectiveness of radiotherapy- and immunotherapy-induced immune activation may be limited, leading to poorer outcomes. This interpretation is supported by previous studies in ESCC (23, 24). A prospective cohort analysis of neoadjuvant chemoradiotherapy plus immunotherapy demonstrated improved pCR and prolonged PFS in patients with higher baseline ALC (13), whereas low ALC has been linked to shorter OS in recurrent/metastatic ESCC treated with ICIs and inferior PFS in postoperative recurrence (25). Collectively, baseline ALC may provide an indication of the host immune reserve, which may be particularly relevant during radiotherapy and immunotherapy. Radiotherapy can enhance antigen presentation, upregulate MHC-I expression, promote T-cell infiltration, and induce immunogenic cell death (26–28), thereby potentially enhancing the efficacy of ICIs. However, radiotherapy does not always have favorable immune effects. Recent evidence suggests that radiotherapy may either support or impair TLS-related antitumor immunity through its effects on lymphocytes and the local immune environment (29). In this context, a higher baseline ALC may represent a greater lymphocyte reserve before treatment. This greater reserve may help maintain antitumor immune responses despite treatment-related lymphocyte loss or immune damage. This may explain why pretreatment ALC was related to clinical outcomes in our study. This may also explain why ALC remained independently related to clinical outcomes after multivariable adjustment, whereas the composite inflammatory ratios did not. These ratios reflect the balance between different blood cell types but do not directly measure circulating lymphocyte levels. In contrast, ALC directly reflects circulating lymphocyte abundance and may serve as a simple and clinically accessible surrogate of host immune status (30, 31). However, this is only a possible explanation. We did not assess lymphocyte subsets, immune markers, TLS status, or changes in ALC during treatment. These mechanisms require further investigation.

The subgroup analyses provided some support for the association between baseline ALC and survival across different treatment groups. However, the interaction results for OS differed between the unadjusted and adjusted analyses of immunotherapy timing. The sequential treatment group was also small. Therefore, this finding needs further confirmation in larger studies.

These findings have two main clinical implications. First, ALC is a routinely available, inexpensive, and easily repeatable laboratory parameter, which makes it a practical indicator in clinical settings. Second, baseline ALC may help identify elderly patients with different clinical outcomes after combined radiotherapy and immunotherapy. Patients with markedly reduced lymphocyte counts may require closer monitoring and a more careful clinical assessment before treatment. Serial assessment of ALC during treatment may also offer more information than a single baseline measurement, although its role in guiding treatment adjustment requires further validation. ALC may also be considered together with other clinical factors for risk stratification in elderly patients.

Nevertheless, this study is subject to several limitations. First, the use of retrospective data from a single institution could have resulted in selection and information bias. Including patients who refused surgery may have increased selection bias. Second, treatment regimens varied. We also did not perform subgroup analysis by radiotherapy dose because of the uneven dose distribution, limited sample size, and lack of a prespecified cutoff for dose stratification. Some important clinical and immune-related factors, such as PD-L1 expression, tumor mutational burden, nutritional status, and comorbidities, were not fully available for analysis. Residual confounding therefore cannot be excluded. Third, we focused on baseline ALC and did not systematically assess changes in ALC during treatment or radiotherapy-related lymphopenia. Fourth, the small non-response group may have made the multivariable logistic model more prone to overfitting. Although the main associations remained significant in the bootstrap analysis, this risk cannot be excluded. The ALC cutoff was derived and assessed in the same cohort, and some variation in the optimal cutoff remained after bootstrap analysis. External validation is therefore needed. Finally, some patients had relatively short follow-up, and treatment-related adverse event data were incomplete. These issues may limit the evaluation of long-term outcomes and treatment safety.

## Conclusion

In conclusion, older patients with inoperable ESCC who received definitive radiotherapy plus immunotherapy showed favorable response and survival outcomes. Baseline ALC was independently associated with short-term treatment response, PFS, and OS. Higher baseline ALC was associated with better clinical outcomes, indicating its potential utility as a simple marker of host immune status. The clinical value of ALC should be further examined in larger prospective multicenter studies.

## Data Availability

The original contributions presented in the study are included in the article/**Supplementary Material**, further inquiries can be directed to the corresponding author/s.
