# Supplementary material for: Association between absolute lymphocyte count and outcomes of radiotherapy combined with immunotherapy in elderly patients with inoperable esophageal squamous cell carcinoma
Source: Front Oncol. 2026 Oct 23;16:1953153. doi: 10.3389/fonc.2026.1953153 (PMC13741178; doi:10.3389/fonc.2026.1953153)
Supplement: Supplementary file 1 [file Table1.docx]

| Outcome | Treatment factor | Subgroup | N | Events, n | HR, 95% CI | *P* | *P* for  interaction | Adjusted *P*  for interaction |
| --- | --- | --- | --- | --- | --- | --- | --- | --- |
| PFS | Chemotherapy regimen | No chemotherapy | 24 | 16 | 0.639 (0.198–2.062) | 0.454 | 0.728 | 0.316 |
|  |  | S-1 | 32 | 22 | 0.591 (0.194–1.798) | 0.354 |  |  |
|  |  | TP | 46 | 29 | 0.304 (0.115–0.799) | 0.016 |  |  |
| OS | Chemotherapy regimen | No chemotherapy | 24 | 14 | 0.460 (0.112–1.888) | 0.281 | 0.603 | 0.670 |
|  |  | S-1 | 32 | 19 | 0.647 (0.193–2.167) | 0.480 |  |  |
|  |  | TP | 46 | 22 | 0.262 (0.086–0.796) | 0.018 |  |  |
| PFS | Immunotherapy timing | Sequential | 20 | 16 | 0.108 (0.018–0.637) | 0.014 | 0.566 | 0.136 |
|  |  | Concurrent | 82 | 51 | 0.479 (0.247–0.929) | 0.029 |  |  |
| OS | Immunotherapy timing | Sequential | 20 | 12 | 0.093 (0.012–0.708) | 0.022 | 0.175 | **0.031** |
|  |  | Concurrent | 82 | 43 | 0.447 (0.213–0.938) | 0.033 |  |  |
| Note: ALC, absolute lymphocyte count; PFS, progression-free survival; OS, overall survival; HR, hazard ratio; CI, confidence interval; PLR, platelet-to-lymphocyte ratio; S-1, tegafur–gimeracil–oteracil; TP, paclitaxel plus cisplatin. ALC was analyzed as a continuous variable. Adjusted P values for interaction were based on models adjusted for tumor stage and PLR. | | | | | | | | |

**Supplementary Table S1.** Subgroup and interaction analyses of baseline ALC and survival outcomes according to treatment characteristics
